# Longitudinal change in hippocampal and dorsal anterior insulae functional connectivity in subjective cognitive decline

**DOI:** 10.1186/s13195-021-00847-y

**Published:** 2021-05-31

**Authors:** Raymond P. Viviano, Jessica S. Damoiseaux

**Affiliations:** 1 Department of Psychology, Wayne State University, 5057 Woodward Ave. 7th Floor Suite 7908, Detroit, MI 48202 USA; 2 Institute of Gerontology, Wayne State University, 87 E. Ferry St, Detroit, MI 48202 USA

**Keywords:** Subjective cognitive decline, Functional connectivity, Longitudinal, Hippocampus, Dorsal anterior insula

## Abstract

**Background:**

Subjective cognitive decline, perceived worsening of cognitive ability without apparent performance issues on clinical assessment, may be an important precursor to dementia. While previous cross-sectional research has demonstrated aberrant brain functional connectivity in subjective cognitive decline, longitudinal evaluation remains limited.

**Methods:**

Here, we examined trajectories of functional connectivity over three measurement occasions ~18 months apart, using voxelwise latent growth models in cognitively unimpaired older adults with varying self-report of subjective cognitive decline (N = 69).

**Results:**

We found that individuals who reported a greater degree of subjective cognitive decline showed a larger subsequent decrease in connectivity between components of the default mode network and increase in connectivity between salience and default mode network components. The change in functional connectivity was observed in the absence of change in cognitive performance.

**Conclusion:**

The results indicate that functional brain changes may underly the experience of cognitive decline before deterioration reaches a level detected by formal cognitive assessment.

**Supplementary Information:**

The online version contains supplementary material available at 10.1186/s13195-021-00847-y.

## Background

Subjective cognitive decline (SCD) is a potential dementia precursor where perceived deterioration of cognitive ability occurs without quantifiable issues on assessment [1]. Recent cross-sectional analyses have identified differences in functional connectivity (FC) between individuals with and without SCD; however, results have been inconsistent. Some groups have reported greater FC within and between default mode network and medial temporal regions while other groups have observed lower FC between these regions or complex patterns of greater and lower FC in SCD [2–12]. The implications of higher default mode network FC in SCD [7–9] are unclear but could reflect compensatory signaling in response to neurodegeneration elsewhere in the brain [13]. However, increased FC may also reflect a shift in network properties unrelated to compensation [14, 15]. Regardless, neurodegenerative pressure could shift elevated FC early in SCD to decreased FC later in SCD. Previous research found lower FC between cortical midline structures in SCD [2, 3], which could represent a later phase of SCD marked by decreased within-network communication. Furthermore, Dillen et al. [6] found that retrosplenial cortex mediated connectivity between hippocampus and default mode network in controls but not in SCD. While involved in episodic memory, cortical midline regions of the default mode network also facilitate self-referential processing [16, 17]. Thus, disruption in the typical communication patterns of these regions could influence perception of the self and contribute to the experience of decline, see Viviano and Damoiseaux [18] for an review on this topic.

The prior results require further analysis for reconciliation but suggest that connectivity aberrations do occur in SCD and that these could represent information processing inefficiencies underlying the perception of decline. The aberrations also occur at network locations commonly implicated in Alzheimer’s disease (AD) and other neurodegenerative diseases [19]. Furthermore, the disparate results could also suggest patterns of non-linear change in FC strength in SCD. It is possible that some research groups find elevated FC in SCD compared to controls while other groups find lower FC in SCD in cross-sectional analysis depending on the sample’s specific phase along the cognitive decline continuum. This possibility highlights the need for longitudinal analysis to determine the characteristics of whole-brain communication patterns in this population.

While most of the extant functional neuroimaging literature in SCD has focused on the default mode network and brain regions associated with memory encoding and retrieval, SCD may affect more than memory networks. Brain regions involved in executive functions [20, 21], and those belonging to a salience network, which send modulation signals to default mode and executive control regions to engage or suppress network activity [22], may also be affected. Indeed, Hu et al. [23] observed lower insula activation in SCD during a task that involved switching between imagination and temporal decision-making, suggesting decreased ability to control engagement of the default mode and executive control networks in SCD. Thus, SCD may relate to inefficient salience network functioning as the dorsal anterior insulae are components of this network [22].

While cross-sectional functional neuroimaging analysis suggests aberrant default mode FC and salience network activity in SCD, longitudinal evaluation of FC remains sparse. Therefore, here, we performed a series of longitudinal analyses to determine resting-state FC change in SCD and how change in FC relates to cognitive performance. We hypothesized that decreased coupling between posterior hippocampus and retrosplenial cortex, and between retrosplenial cortex and medial prefrontal cortex would occur over time in SCD, reflecting a breakdown in communication of posterior default mode network components important for episodic recollection [24]. We also hypothesized that the dorsal anterior insulae would decouple from executive control and default mode network regions in SCD; possibly reflecting decreased ability to modulate network activity.

Next, we evaluated how the experience of cognitive decline relates to trajectories of cognitive performance change. While older adults with SCD test within the normal range on cognitive assessment per the definition [25], in practice, this determination is sometimes made with brief clinical assessments, such as the Mini-Mental State Exam (MMSE) [26]. Meta-analytic evaluation has demonstrated that, on average, older individuals with SCD perform poorer than older individuals without SCD, though the effect size is small [27]. Therefore, individuals with SCD exhibit subtle decline in aggregate. In addition, as older adults with SCD are more likely to develop dementia [28, 29], they may also decline at a faster rate than individuals without cognitive concerns. Thus, longitudinal evaluation may detect potential differences in their cognitive trajectory. A mediation of FC change on the relationship between SCD and cognitive change could indicate that inefficient whole-brain network processing underlies the progression of subjective decline to objective decline. For the present analysis, we anticipated a decrease in working memory and delayed memory performance in SCD. In addition, we hypothesized that decreased FC between salience and executive control network regions mediates the relationship between SCD and performance on working memory tasks, and that decreased coherence between default mode regions and hippocampus predicts decreased delayed memory performance.

## Methods

### Participants

Baseline magnetic resonance imaging, demographic, and cognitive assessment data were available from 69 adults (ages range from 50 to 85 years, *M* = 68.33, *SD* = 7.95) from Metro Detroit that participated in a broader multi-site study evaluating brain structure and function differences between healthy older adults with and without SCD (see [3] for more information). Of the 69 participants, 49 were enrolled in the longitudinal arm of the study. For these participants, data collection occurred approximately every 1.5 years (*M* = 18.82 months, *SD* = 1.42) for three measurement periods. Of these 49 participants, 34 returned for the second measurement (70% response rate), while 28 returned for the third measurement (57% response rate). As indicated above, only baseline data was available for the remaining 20 participants. All participants were right-handed (Edinburgh Handedness *M* = 96.32, *SD* = 7.61), reported no psychiatric or neurologic disorders, reported no prior head trauma, spoke English as a native language, and had no magnetic resonance imaging contraindications. Individuals provided informed consent prior to participation at each measurement occasion. The Wayne State University Institutional Review Board approved the data collection procedures for this study. Demographic and cognitive performance information is available for the full sample in Table 1.

**Table 1:** Demographic and cognitive metrics for all participants across three timepoints. For continuous variables, values in the cells represent means ± standard deviations. MFQ-FoF: Memory Functioning Questionnaire Frequency of Forgetting Inverted Average; MMSE: Mini-Mental State Examination; WASI: Wechsler Abbreviated Scale of Intelligence; WMS-IV: Wechsler Memory Scale IV

	Time 1 (N = 69)	Time 2 (N = 34)	Time 3 (N = 28)
Time between measurements (months)	-	19.10 ± 1.62	18.43 ± .96
Baseline age (years)	68.33 ± 7.95	67.41 ± 8.95	66.46 ± 9.52
Sex (female, male)	56 F, 13M	29 F, 5 M	24 F, 4 M
Racial identity (African American/white)	54 / 15	25 / 9	20 / 8
Doctor seen for memory concerns	19 Y, 50 N	10 Y, 25 N	3 Y, 25 N
Family history of Alzheimer’s disease	34 Y, 35 N	19 Y, 15 N	11 Y, 17 N
Geriatric Depression Scale (GDS)	3.82 ± 3.93	3.62 ± 3.70	3.71 ± 3.71
Beck Depression Inventory	4.59 ± 4.12	6.71 ± 5.49	5.56 ± 3.66
Big Five Conscientiousness	37.36 ± 5.48	37.35 ± 4.60	36.75 ± 4.56
Big Five Neuroticism	17.41 ± 6.00	17.29 ± 5.39	17.89 ± 5.74
MFQ-FoF	2.99 ± .91	2.95 ± .95	2.91 ± .79
MMSE	28.32 ± 1.97	28.83 ± 1.16	28.86 ± 1.30
WASI Full IQ 4-Scale	98.09 ± 11.45	101.87 ± 12.93	100.96 ± 13.03
Rey Auditory Total	46.43 ± 9.31	48.90 ± 8.87	47.64 ± 10.03
WMS-IV Auditory Index	.47 ± .12	.55 ± .11	.54 ± .13
WMS-IV Visual Index	.54 ± .11	.55 ± .12	.57 ± .15
WMS-IV Visual Working Memory Index	.40 ± .12	.42 ± .11	.41 ± .15
WMS-IV Immediate Memory Index	.55 ± .10	.58 ± .10	.58 ± .12
WMS-IV Delayed Memory Index	.47 ± .11	.51 ± .12	.53 ± .14
Trail making task B/A ratio	2.64 ± 1.25	2.34 ± .84	2.13 ± .87
Digit symbol substitution total	39.78 ± 11.41	41.70 ± 10.89	43.86 ± 13.06
Stroop task ratio	2.00 ± .47	1.91 ± .30	1.96 ± .41
Verbal fluency total	36.55 ± 7.46	39.74 ± 7.82	38.04 ± 8.79

### Magnetic resonance imaging data collection

Magnetic resonance imaging data collection occurred on a 3-Tesla Siemens Magnetom Verio full-body magnet (Siemens Medical AG, Erlangen, Germany) with a 32-channel head coil, located at the Wayne State University Magnetic Resonance Research Facility. The structural image available for functional image co-registration was a 3D T1-weighted magnetization-prepared rapid gradient-echo (MP-RAGE) sequence with 176 slices collected parallel to the bicommissural line. Repetition time (TR) = 1680 ms, echo time (TE) = 3.51 ms, inversion time = 900 ms, flip angle = 9.0°, pixel bandwidth = 180 Hz/pixel, GRAPPA acceleration factor PE = 2, field of view (FOV) readout = 256 mm, FOV phase = 100%, matrix size = 384 × 384, voxel size = 0.67 × 0.67 × 1.34 mm. A high-resolution multiband T2*-weighted echoplanar functional image was available for the FC analyses with 75 slices parallel to bicommissural line, 220 image volumes, TR = 2000 ms, TE = 30 ms, flip angle = 73°, pixel bandwidth = 1698 Hz/pixel, GRAPPA acceleration factor PE = 2, multiband acceleration factor = 3, FOV readout = 256 mm, FOV phase = 100%, matrix size = 128 x 128, and voxel size = 2.00 mm isotropic. Participants kept their eyes closed for the resting-state procedure. Two spin-echo echoplanar images with similar parameters to the resting-state multiband image and opposing phase encoding directions (anterior to posterior and posterior to anterior) were acquired to compute a field map for distortion correction. Parameters: 75 slices parallel to the bicommissural line, 3 image volumes, TR = 2416 ms, TE = 51 ms, echo spacing = .69 ms, flip angle = 90°, pixel bandwidth = 1698 Hz/pixel, GRAPPA acceleration factor PE = 2, multiband acceleration factor = 3, FOV readout = 256 mm, FOV phase = 100%, matrix size = 128 × 128, and voxel size = 2.00 mm isotropic.

### Subjective cognitive decline evaluation

We evaluated SCD as a continuous variable with the Memory Functioning Questionnaire Frequency of Forgetting (MFQ-FoF) subscale [30]. This subscale query participants on how frequently they feel that remembering names, directions, dates, etc. are problematic. All items are on a 7-point Likert scale with 1 representing frequent concern and 7 representing no concern. For ease of interpretability, we inverted the Likert scoring so that larger numbers reflected a greater degree of SCD and then took the average across questions for each individual. Then, we subtracted one so that the range of possible scores would be between zero and six, ensuring more interpretable intercept terms in the latent growth models. We evaluated MFQ-FoF at baseline only rather than as a time-varying covariate in our statistical analyses as there was no appreciable overall change in the measure over the 3-year data collection period, determined with a mixed effects model (Supplementary Table 1)—detailed descriptive statistics for MFQ-FoF in Supplementary Table 2.

### Objective cognitive function evaluation

All participants either scored ≥ 25 on the MMSE, considered within the normal range [31], or had a recent clinical consensus as cognitively normal. Note that all participants ≤ 70 years-of-age scored ≥ 27 on the MMSE. Furthermore, all participants performed in the cognitively normal range at baseline, defined as performance no worse than two standard deviations below the normative mean on any two Wechsler Memory Scale IV (WMS-IV) indices [32]. This somewhat lenient cutoff was used to minimize the risk for misclassifying cognitive unimpaired as impaired based on the following rationales: first, all of our participants completed the adult battery of the WMS-IV rather than the older adult battery to keep data collection consistent across participants. The adult battery only provides normative scores up to age 69. Thus, normative performance derivation for an 80-year-old required comparison to 69-year-old normative performance, which deflated the measures for many participants. Second, we aimed to mitigate the elevated risk of misclassifying cognitively normal older adults as impaired when they complete multiple tests as was the case here [33, 34]. Third, but not least, our dataset consists of largely African American participants. It is well-established that cognitively unimpaired African American adults tend to score lower on neuropsychological tests than cognitively unimpaired white adults [35, 36]. When there are no culturally appropriate norms available there is a risk of interpreting their scores as below average while they likely are not, which we aimed to prevent here. In all our statistical models, we used the raw cognitive test scores.

We evaluated executive functioning and memory performance with the Visual Working Memory and Delayed Memory indices of the WMS-IV [32]. Two subtasks comprise the Visual Working Memory Index: Spatial Addition and Symbol Span. The Spatial Addition task requires mental manipulation of objects in space, inhibition of irrelevant stimuli, and maintenance of information which are all components of the broader category of executive functioning. The symbol span task requires the maintenance of abstract stimuli and their ordering over a short period of time and thus queries working memory capabilities. The Delayed Memory Index of the WMS-IV includes the delayed administrations of the Visual Reproduction, Logical Memory, Verbal Paired Associates, and Design Memory subtasks. Thus, the delayed memory composite score summarizes auditory, visual, logical, and spatial long-term memory.

### Resting-state functional magnetic resonance imaging processing

We used the FMRIB Software Library FEAT pipeline to process the multiband resting-state functional imaging data, which included removal of the first five image volumes to account for early field inhomogeneities, motion correction [37], non-brain structure removal [38], susceptibility-based distortion correction using the field map generated from the spin-echo echoplanar images [39, 40], co-registration to the anatomical image with boundary-based registration, and subsequent registration to Montreal Neurologic Institute 2-mm standard space with a 12 degree of freedom affine transformation [41], spatial smoothing (4 mm FWHM), and 4D-grand-mean-scaling. We treated each timepoint as independent when registering to standard space, which results in low bias at the tradeoff of high variance [42]. Furthermore, we used ICA-AROMA, an independent component analysis-based method to detect and regress motion artifact components from functional images [43], to remove structured noise. We also regressed global signal from the images. Framewise displacement statistics are available in Supplementary Table 4.

Next, we generated whole-brain seed-based FC maps from hippocampal, retrosplenial, and dorsal anterior insulae seeds (Table 2), with coordinates derived from previous analyses or meta-analyses [44–46] and converted from Talairach to Montreal Neurologic Institute space when necessary [47]. We chose to evaluate posterior hippocampal and retrosplenial cortex seeds due to involvement of these regions in memory retrieval [24]. Furthermore, we evaluated dorsal anterior insulae seeds as these regions are core to the salience network [22] and we hypothesized that disruption between the salience network regions and either the default mode or executive control networks would occur over time in SCD. We chose hippocampal coordinates that corresponded to the hippocampal body [45] as posterior regions of the hippocampus (body and tail) connect directly and indirectly to posterior default mode network regions and are involved in memory retrieval [24, 48]. In addition, we shifted the retrosplenial coordinate anteriorly by two voxels to avoid potential signal contamination from the precuneus. We computed Fisher Z-Transformed Pearson correlations between the average signal from 12-mm diameter spherical regions-of-interest, masked with a cortical gray matter mask, with the signal from every other gray matter voxel to produce seed-based correlation maps for each participant at every timepoint.

**Table 2:** Region-of-interest central coordinates in MNI space

Region	X	Y	Z	Citation
Retrosplenial cortex	2	−48	24	[44]
Left posterior hippocampus	−26	−30	−10	[45]
Right posterior hippocampus	26	−30	−10	[45]
Left dorsal anterior insula	−32	20	2	[46]
Right dorsal anterior insula	36	20	−2	[46]

### Functional connectivity conditional latent growth models

To evaluate change in FC in SCD, we used Neuropointillist [49] to fit conditional latent growth curve models to the voxelwise, seed-based connectivity maps, using full information maximum likelihood estimation (FIML). Neuropointillist is an R library for processing statistical models, including structural equation models, at the voxel level in parallel on server clusters. We centered the latent intercept at the first timepoint and measured linear change with the latent slope. We evaluated the relationship between FC change and MFQ-FoF while controlling for baseline age centered at 70. There were negative residual variances for parameters of some voxels. However, these were small, and the 95% confidence intervals contained positive values. We interpreted these as resulting from sampling error due to small sample size. For voxels where this was an issue, we constrained negative residual variance terms to 0 and reran the models if the original confidence interval contained 0. This made the model slightly different across voxels, but in practice only affected a subset. We only report results from voxels where we did not apply constraints.

**Fig. 1:**
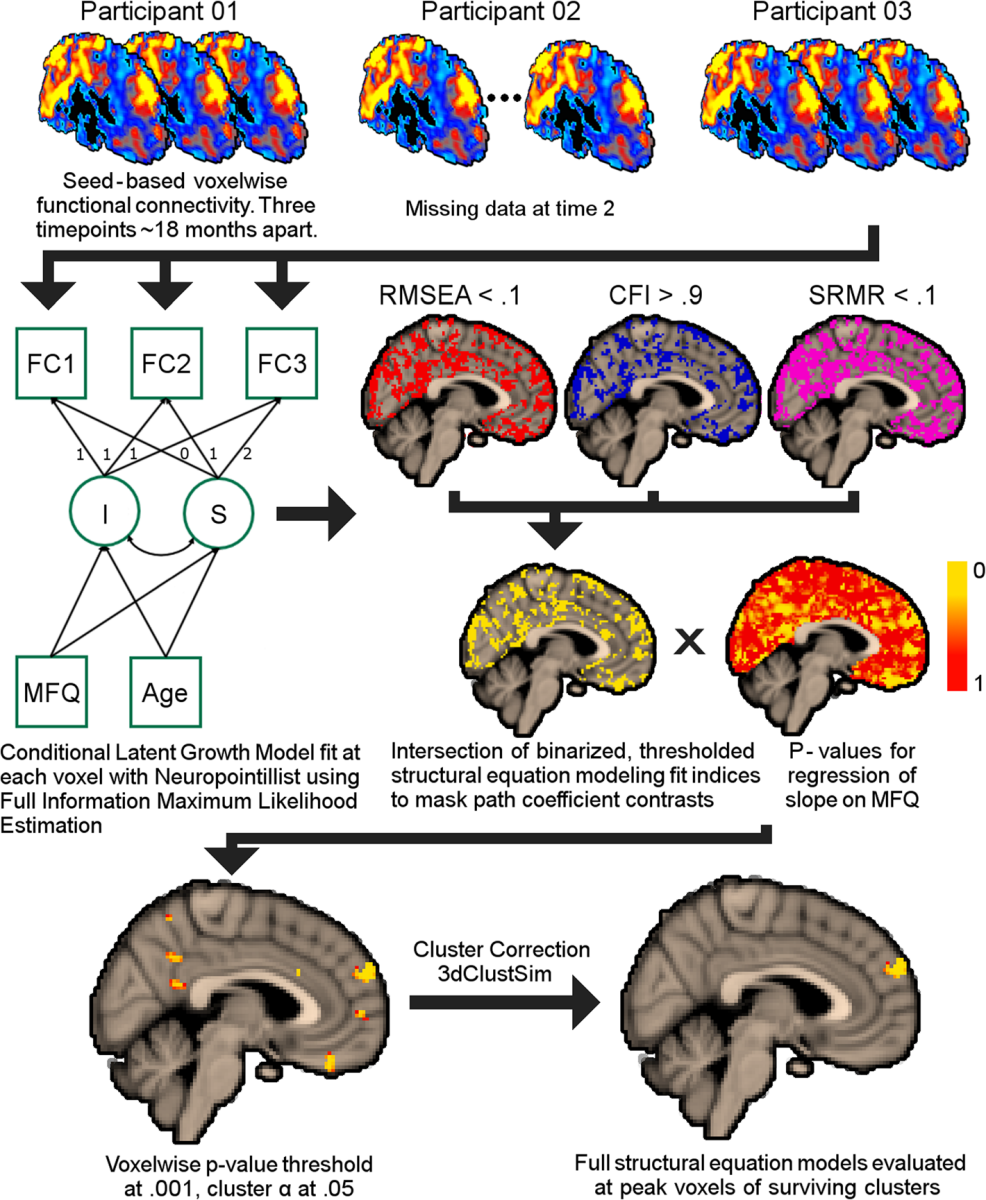
Analytic approach to longitudinal functional connectivity analysis. Here, we fit conditional latent growth models at the voxel level of seed-based correlation images with full information maximum likelihood estimation, which is robust to data missing at random. After masking for reasonable structural equation model fit and cluster correction, we evaluated models at clusters where there was a significant effect of MFQ-FoF on FC change

Neuropointillist creates 3D images separately for fit indices, path coefficients, variances, and p values. We focused on the regression of FC slope on MFQ-FoF images to identify locations where the conditional models fit well, and there was a significant association between degree of cognitive concern and change in FC. Specifically, we masked the regression of FC slope on MFQ-FoF images with a binary mask reflecting reasonable model fit, where 1 indicated Root Mean Square Error of Approximation (RMSEA) ≤ .10, Comparative Fit Index (CFI) ≥ .90, and Standardized Root Mean Square Residual (SRMR) ≤ .10. Then, we extracted clusters that survived multiple comparison correction, *p* < .001, cluster α < .05, 240 mm^3^ minimum cluster extent [50]. For clusters that survived the voxel count cutoff, we report the models for the voxel in the cluster where the parameter estimate for regression of slope on MFQ-FoF was at peak and where we did not have to constrain any residual variances to 0. We also fit latent growth models without covariates (i.e., unconditional models) to evaluate interindividual variability in FC change for clusters that survived multiple comparison correction. The results of these unconditional models inform us on the extent of individual differences in FC change and thereby provide context for the effect of SCD (as measured by the MFQ-FoF) in explaining these differences. We interpreted model parameters from clusters of interest at the *p* < .01 level to account for the five ROIs examined. See Fig. 1 for analytic approach.

### Models for functional connectivity and cognitive performance change

**Fig. 2:**
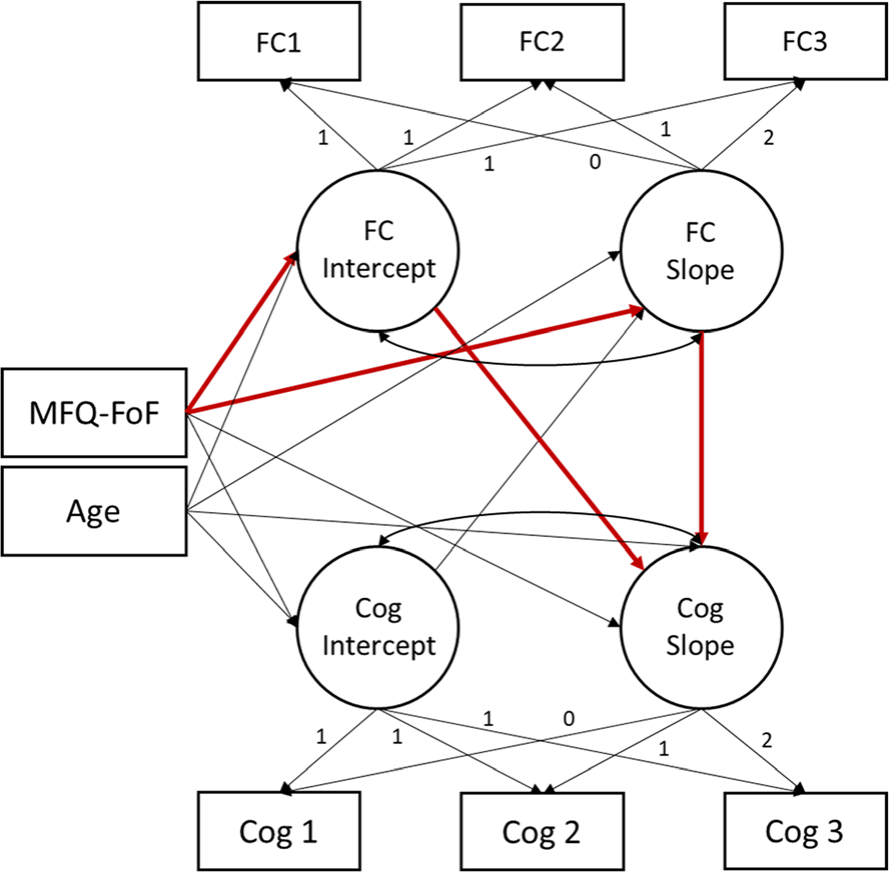
Diagram of functional connectivity and cognitive performance parallel process model fit at each voxel individually. Indirect paths of interest displayed in red, though we did not evaluate potential mediation effects as we did not identify any significant clusters. Abbreviations: functional connectivity (FC), cognitive performance (Cog), Memory Functioning Questionnaire Frequency of Forgetting Subscale (MFQ-FoF)

To evaluate the potential for change in FC to mediate the association between MFQ-FoF and change in cognitive performance, we employed parallel process models with FIML at the voxel level similar to the FC conditional latent growth models. For these models, we regressed the slope of change in cognitive performance on the intercept and slope terms of the FC growth process while regressing all latent terms on MFQ-FoF and age (Fig. 2). We evaluated working memory performance with left and right dorsal anterior insula FC and delayed memory performance with left and right hippocampal FC. Before evaluating the parallel processes of FC and cognitive performance change, we first evaluated if there was change, and variability in change, in cognitive performance and if SCD related to these growth processes with conditional latent growth models. Though we did not discover change in either delayed memory or working memory performance related to SCD, we still tested the voxelwise parallel process to evaluate if FC latent growth processes related to change in cognitive performance controlling for MFQ-FoF and age. We planned to estimate if FC growth processes mediated the effect of SCD on change in cognitive performance. However, as we did not discover significant clusters where growth processes in FC related to change in cognitive performance, we could not perform such mediation analyses.

### Data missingness

With longitudinal analysis, attrition may bias results. Therefore, we performed a series of univariate logistic regressions to determine if baseline measures related to lack of follow-up data at timepoints 2 or 3. Baseline age was marginally related to missingness at timepoint 3 (*OR* = 1.04, *p* = .07); however, as the odds ratio was close to 1 and the p value was above a .05 cutoff, we interpreted the missingness mechanism as missing at random. Nevertheless, by including age as a covariate in subsequent models, we accounted for potential missingness related to this variable as a source of variance and converted the data missingness mechanism to missing at random when FIML was applied, which is robust to this level of missingness, and reduced potential bias in parameter estimates. No other variable related to missingness (*p* ≫ .20).

## Results

### Demographics

There were more women in the sample at baseline than men, 81%, *χ*^*2*^*(1)* = 26.80, *p* < .01. Because there were significantly more women in the sample than men, and because the number of men in the sample beyond the first timepoint was ≤ 5, we did not assess sex as a covariate of change. The sample was also predominantly African American, 78%, *χ*^*2*^*(1)* = 22.04, *p* < .01, which differentiates this dataset from many other healthy aging and pre-dementia datasets with predominantly white samples. A summary of demographic and cognitive metrics for the full sample at every timepoint is available in Table 1. MFQ-FoF was unrelated to MMSE at baseline and did not predict change in MMSE (Supplementary Table 3).

### Functional connectivity latent growth models

#### Left posterior hippocampus functional connectivity

**Fig. 3:**
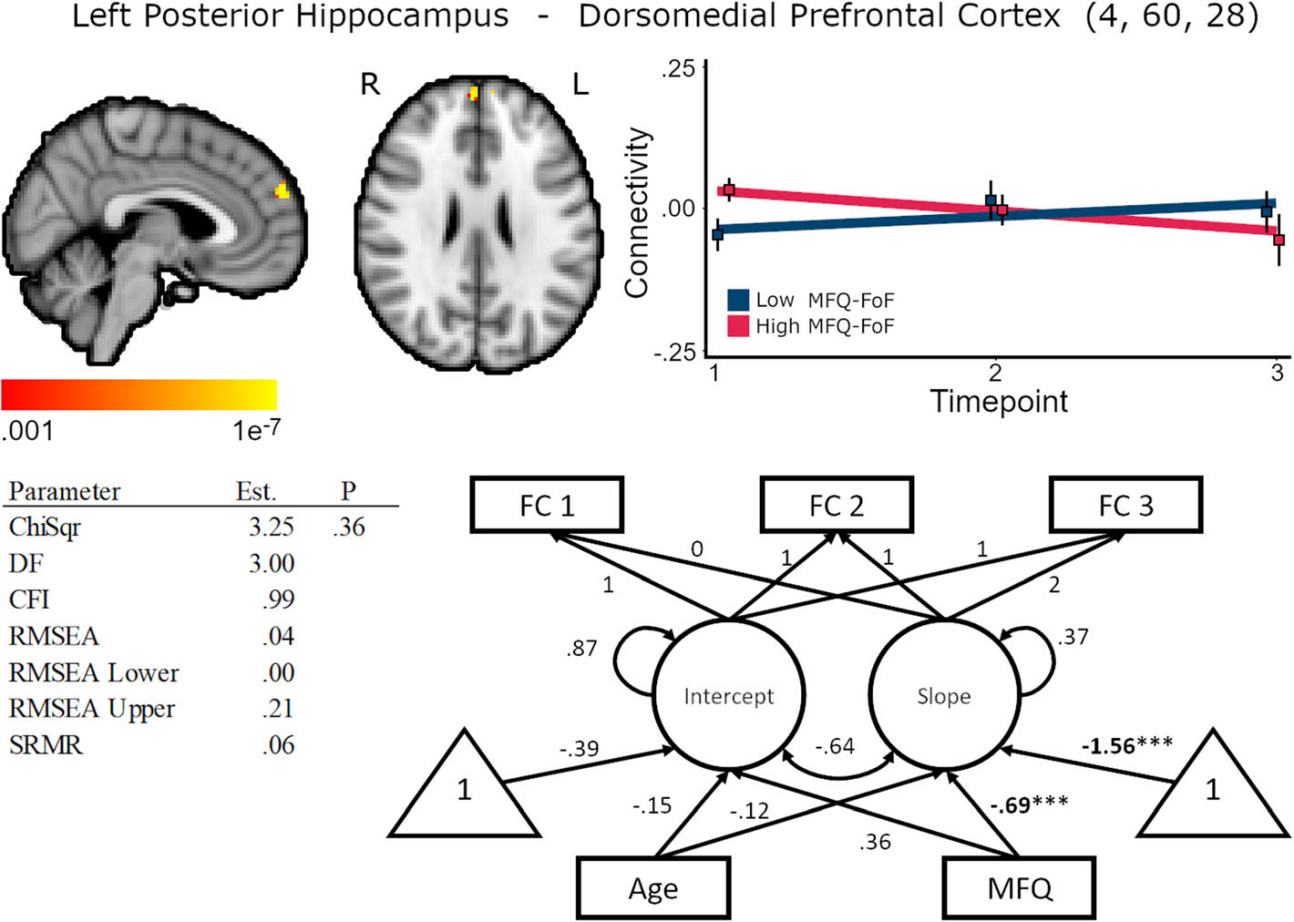
Baseline MFQ-FoF predicted decreasing connectivity between the left hippocampus and dorsomedial prefrontal cortex, 248 mm^3^ cluster size. Brain image cluster gradients reflect p values between .001 and 1e−7. Fit lines for FC trajectory based on median split of MFQ-FoF values with the red reflecting the mean trajectory for older adults with a greater degree of SCD at baseline and blue reflecting individuals with fewer baseline concerns. This median split was only for visualization and is not reflected in the structural equation models. Model fit and parameter estimates are for the peak voxel

Higher MFQ-FoF was associated with decreasing FC between left hippocampus and dorsomedial prefrontal cortex, standardized coefficient = −.69, *p* < .01, but was not associated with baseline FC, standardized coefficient = .36, *p* = .03, (Fig. 3, Supplementary Table 5). As connectivity between the hippocampus and dorsomedial prefrontal cortex may be important for long-term memory processes [51], decreasing connectivity between these regions may be sufficient for the perception of cognitive decline. Participants' age was unrelated to baseline FC, standardized coefficient = −.15, *p* = .50, and unrelated to change in FC, standardized coefficient = −.12, *p* = .51. Latent slope variances across the voxels of the cluster ranged between <.001 and .009, .004 ≤ *p* ≤ .999, for the unconditional model. Therefore, individual differences in FC change across the cluster were small; however, MFQ-FoF explained a significant amount of the variability in FC change.

**Fig. 4:**
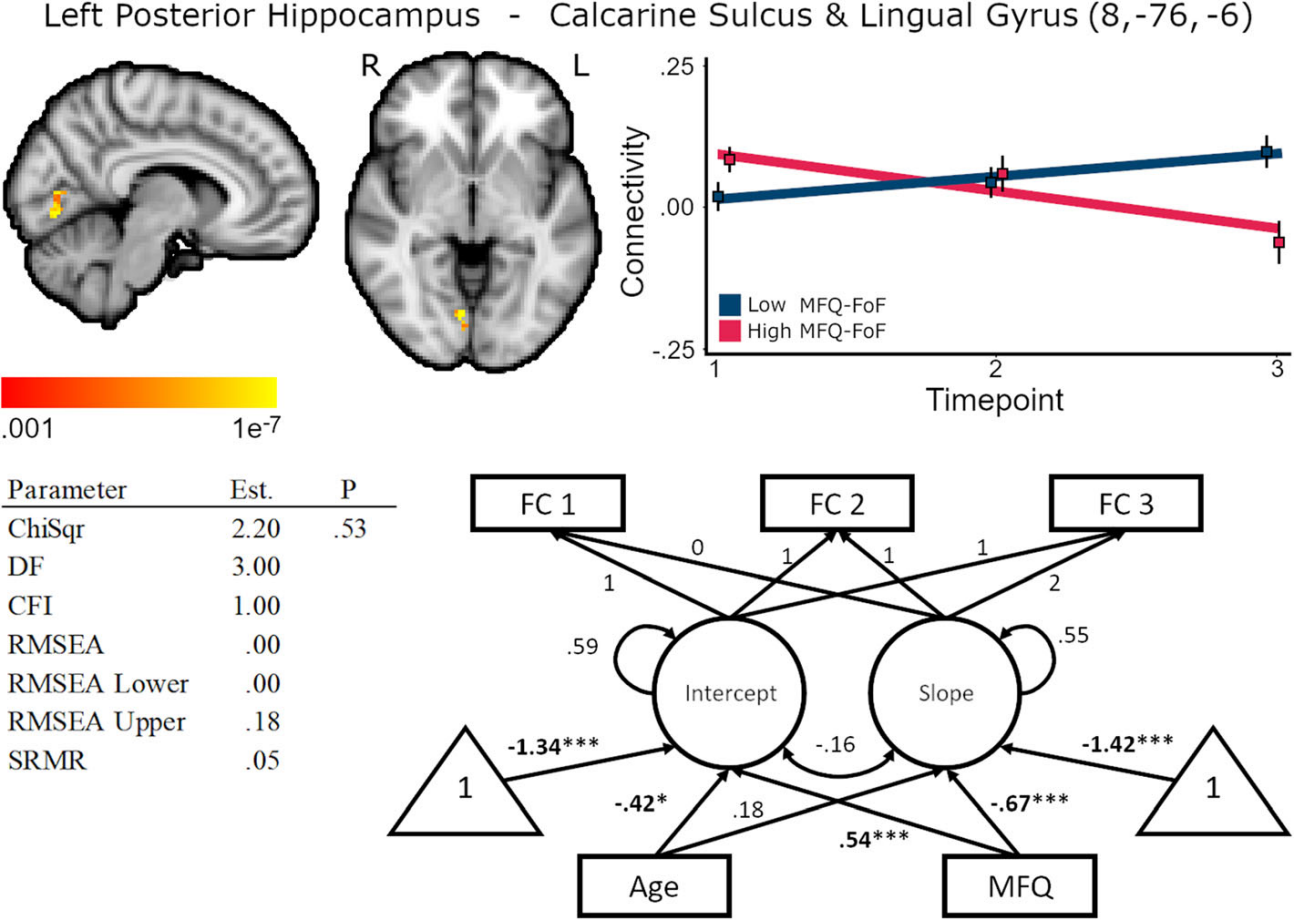
Baseline MFQ-FoF predicted decreasing connectivity between the left hippocampus and calcarine sulcus and lingual gyrus, 584 mm^3^ cluster extent. Brain cluster gradients reflect p values between .001 and 1e−7. Fit lines for FC trajectory based on median split of MFQ-FoF values with the red reflecting the mean trajectory for older adults with a greater degree of SCD at baseline and blue reflecting individuals with fewer baseline concerns. This median split was only for visualization and is not reflected in the structural equation models. Model fit and parameter estimates are for the peak voxel

Higher MFQ-FoF was also associated with decreasing connectivity between the left hippocampus and lingual gyrus/calcarine sulcus, standardized coefficient = −.67, *p* < .01, and was associated with greater baseline FC between those regions, standardized coefficient = .54, *p* < .01, (Fig. 4, Supplementary Table 5). It is possible that decreasing FC between these regions could reflect disrupted flow of visual information to the hippocampus which could in turn affect visual memory processing and influence perceived decline. This result also strengthens support for SCD as a dementia precursor as there may be an Alzheimer’s disease subtype distinguished by pronounced visual cortex atrophy [52]. Older age was associated with lower baseline FC, standardized coefficient = −.42, *p* = .01, but was unrelated to FC change, standardized coefficient = .18, *p* = .26. Latent slope variances were between < .001 and .004, .18 ≤ *p* ≤ .99, for the unconditional model.

#### Right posterior hippocampus functional connectivity

**Fig. 5:**
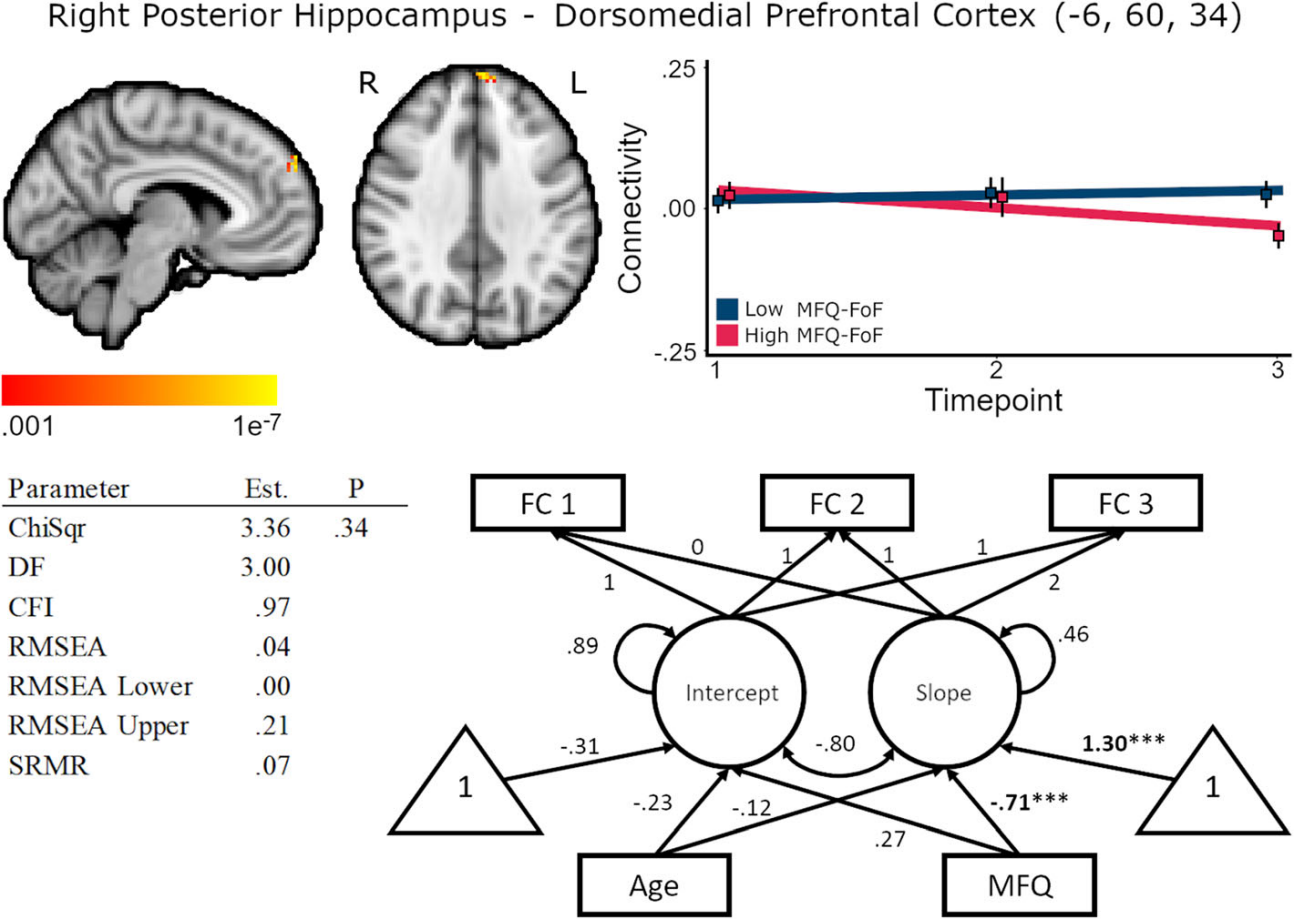
Baseline MFQ-FoF predicted decreasing connectivity between the right hippocampus and dorsomedial prefrontal cortex, 312 mm^3^ cluster size. Brain image cluster gradients reflect p values between .001 and 1e−7. Fit lines for FC trajectory based on median split of MFQ-FoF values with the red reflecting the mean trajectory for older adults with a greater degree of SCD at baseline and blue reflecting individuals with fewer baseline concerns. This median split was only for visualization and is not reflected in the structural equation models. Model fit and parameter estimates are for the peak voxel

Higher MFQ-FoF was associated with decreasing FC between the right hippocampus and dorsomedial prefrontal cortex, standardized coefficient = −.71, *p* < .01, but was not associated with baseline FC, standardized coefficient = .27, *p* = .09 (Fig. 5, Supplementary Table 5). As with the observation of decreasing connectivity between the left hippocampus and dorsomedial prefrontal cortex, this result also suggests that disruption in long-term memory processing mediated by hippocampal to prefrontal connectivity could be an important influence on the perception of declining cognitive ability. Furthermore, similar results of decreasing connectivity to the medial prefrontal cortex across lateralized evaluation of hippocampal connectivity suggests that this may be a robust feature of SCD. Age was not associated with baseline FC, standardized coefficient = −.23, *p* = .14, nor change in FC, standardized coefficient = −.12, *p =* .54. Latent slope variances across the cluster were between < .001 and .004, .14 ≤ *p* ≤ .87, for the unconditional model.

#### Left dorsal anterior insula functional connectivity

**Fig. 6:**
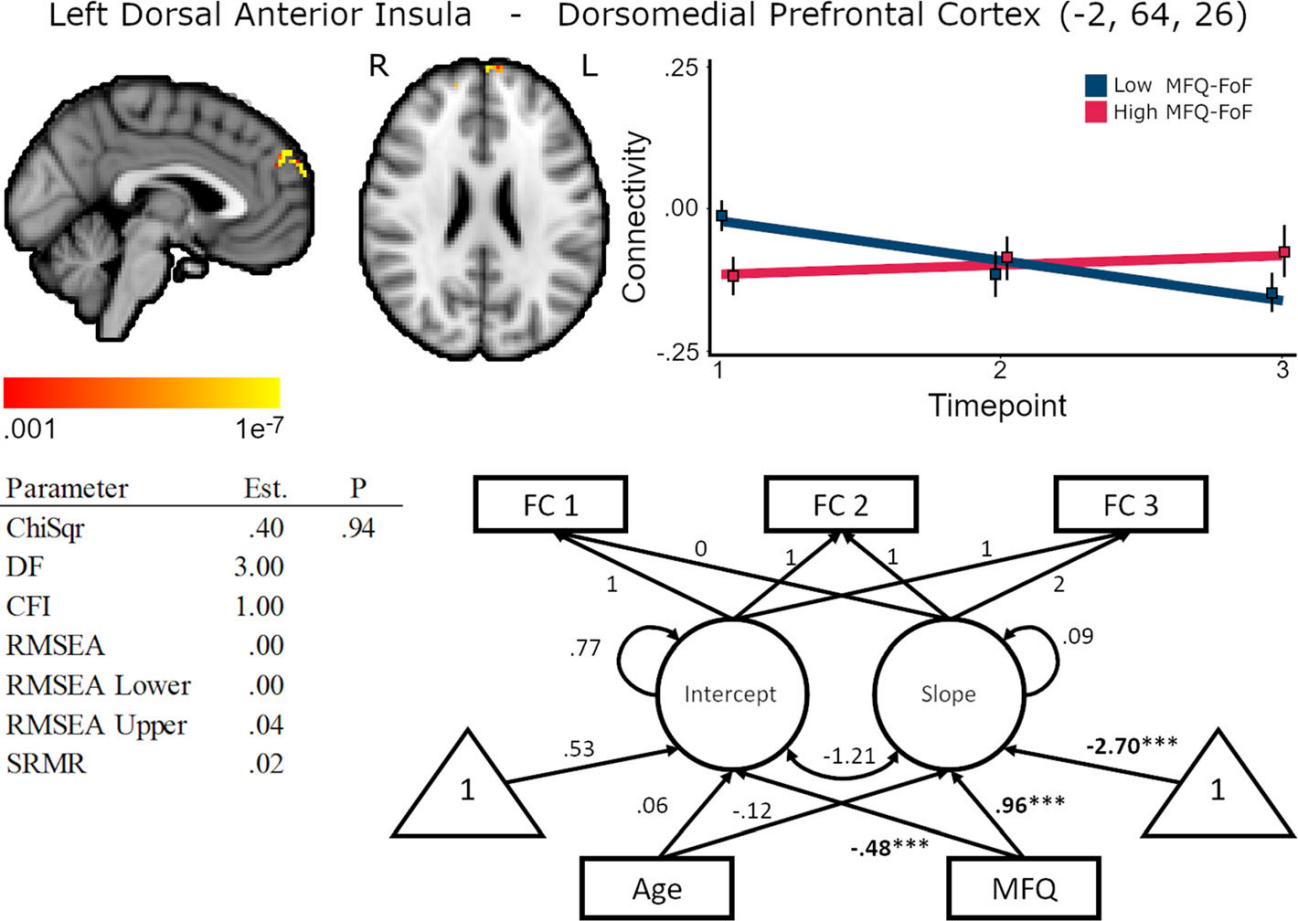
Baseline MFQ-FoF was positively associated with the left dorsal anterior insula and dorsomedial prefrontal cortex FC slope, 352 mm^3^ cluster size. Brain image cluster gradients reflect p values between .001 and 1e−7. Fit lines for FC trajectory based on median split of MFQ-FoF values with the red reflecting the mean trajectory for older adults with a greater degree of SCD at baseline and blue reflecting individuals with fewer baseline concerns. This median split was only for visualization and is not reflected in the structural equation models. Model fit and parameter estimates are for the peak voxel

MFQ-FoF was positively associated with left dorsal anterior insula and dorsomedial prefrontal cortex FC latent slope, standardized coefficient = .96, *p* < .01, and was also associated with lower baseline FC, standardized coefficient = −.48, *p* < .01, (Supplementary Table 5). Plotting trajectories of FC change by MFQ (Fig. 6) revealed that individuals with a higher degree of SCD had lower connectivity at baseline that increased slightly over the measurements but remained relatively stable, while individuals with a lower degree of SCD had greater connectivity between regions initially that decreased over time. This could indicate that there is a floor effect that is individuals with a greater degree of SCD are already at the lower bounds of connectivity between these regions for healthy aging. Lower connectivity between the dorsal anterior insula and dorsomedial prefrontal cortex could be a feature of converting to objective cognitive decline for these individuals. Individuals with lower baseline SCD may have more leeway to exhibit connectivity declines between these regions while maintaining stable cognitive performance. Age was not associated with baseline FC, standardized coefficient = .06, *p* = .68, nor change in FC, standardized coefficient = −.12, *p* = .67. Latent slope variances across the cluster were between <.001 and .006, .16 ≤ *p* ≤ .99, for the unconditional model.

**Fig. 7:**
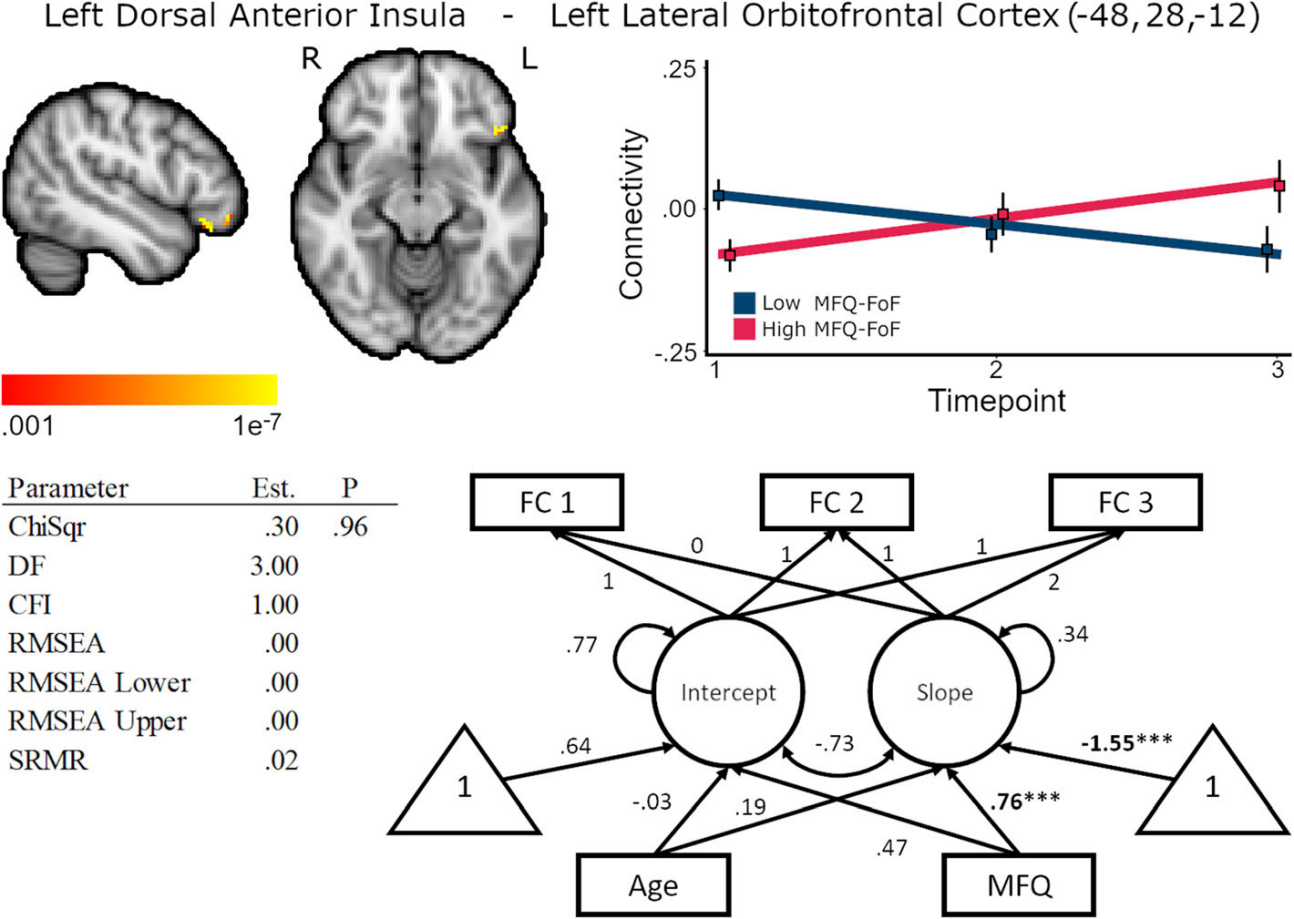
Baseline MFQ-FoF predicted increasing connectivity between the left dorsal anterior insula and left lateral orbitofrontal cortex, 248 mm^3^ cluster size. Brain image cluster gradients reflect p values between .001 and 1e−7. Fit lines for FC trajectory based on median split of MFQ-FoF values with the red reflecting the mean trajectory for older adults with a greater degree of SCD at baseline and blue reflecting individuals with fewer baseline concerns. This median split was only for visualization and is not reflected in the structural equation models. Model fit and parameter estimates are for the peak voxel

Higher MFQ-FoF was also associated with increasing connectivity between the left dorsal anterior insula and left lateral orbitofrontal cortex, standardized coefficient = .76, *p* < .01, but not with baseline FC, standardized coefficient = −.47, *p* = .02 (Fig. 7, Supplementary Table 5). This could indicate aberrant signaling changes between salience network regions and regions important for executive functions occur in SCD. Age was not associated with baseline FC, standardized coefficient = −.03, p = .86, nor change in FC, standardized coefficient = .19, p = .26. Latent slope variances across the cluster were between <.001 and .011, *p* ≤ .01, for the unconditional model.

#### Right dorsal anterior insula and retrosplenial cortex functional connectivity

There were no clusters where MFQ-FoF explained a significant amount of FC latent slope variance that survived the multiple comparison correction cutoff for the right dorsal anterior insula and retrosplenial cortex models.

### Cognitive performance and functional connectivity

The initial conditional latent growth models for the WMS Visual Working Memory Index had a negative disturbance for latent slope. As the 95% confidence interval contained 0, we constrained this term to 0 (Supplementary Table 6). For the constrained model: *χ*^*2*^*(3)* = 2.77, *p* = .60, CFI = 1.00, RMSEA = .00, RMSEA 95% CI = [.00, .16], SRMR = .03. There was significant interindividual variability in working memory intercept, variance *=* .01, *p* < .01, for the unconditional model. However, latent slope variability was unevaluable due to the model constraint. For the full model, MFQ-FoF was unrelated to change in performance. However, older age was associated with poorer baseline working memory performance, standardized coefficient = −.62, *p* < .01, though age was not associated with change in performance, standardized coefficient = −.67, *p* = .49.

The WMS Delayed Memory Index latent growth models did not require model constraints (Supplementary Table 7). Overall model fit was poor, *χ*^*2*^*(3)* = 4.35, *p* = .23, CFI = .99, RMSEA = .08, confidence interval = [.00, .22], SRMR = .06. There was significant variance in the latent intercept term, variance = .01, *p* < .01, for the unconditional model. However, latent slope variance was not significant for the unconditional model, variance < .01, *p* = .57. For the full model, MFQ-FoF was unrelated to delayed memory performance. But older age associated with poorer performance at baseline, standardized coefficient = −.50, *p* < .01; and with decreasing delayed memory performance, standardized coefficient = −.32, *p* < .01.

### Functional connectivity and cognitive performance parallel process models

For the parallel process models, there were no clusters where FC growth processes explained a significant amount of cognitive performance latent slope variance that survived cluster-level multiple comparison correction. We could not identify meaningful locations to extract model parameters to report. Therefore, there was no indication that baseline hippocampal FC, nor change in FC, mediated any association between MFQ-FoF and change in cognitive performance.

## Discussion

The present analyses evaluated longitudinal FC and cognitive trajectories in older adults that harbored varying degrees of subjective cognitive decline. Our results show that the degree of SCD predicts FC change within and between nodes of the default mode and salience networks. As these brain changes occurred without concomitant cognitive changes, the results could indicate that brain changes underly the perception of decline and could be a sensitive marker for nascent dementia before assessment can detect cognitive deficit.

The presence of more SCD predicted decreasing FC between the dorsomedial prefrontal cortex and both left and right posterior hippocampus. We interpret this as decreasing connectivity within the default mode network. Different sections of the dorsomedial prefrontal cortex are included in the executive control and default mode networks during network analyses [21, 53] with the more anterior dorsomedial prefrontal cortex, which the present clusters were located at, belonging to the default mode network [54]. The extant literature suggests that this region of cortex is associated with higher-level abstract processing of social and non-social information [55, 56], theory of mind, and long-term memory [57]. Though dorsomedial prefrontal cortex serves many functions and disrupted hippocampal communication to this region could affect many processes, decreased coherence between these regions could reflect disruption in long-term memory processing and social cognitive functioning as medial temporal connectivity to rostral segmentations of the dorsomedial prefrontal cortex may be important to these processes specifically [51]. This may influence the self-perception of memory and social abilities relative to peers and could be sufficient for the perception of cognitive decline. Decreasing the dorsomedial prefrontal to posterior hippocampal FC also indicates that within-default mode FC alterations are robust attributes of SCD and fits in with the extant cross-sectional literature that has identified default mode FC differences between older adults with and without SCD [2, 3, 8, 9]. Furthermore, decreased default mode network connectivity in patients with mild cognitive impairment and Alzheimer’s disease is consistently found [58–60]. The present results support SCD as a stage where a greater rate of FC change could be a factor that distinguishes older individuals at greater risk of cognitive decline from those that will maintain stable cognitive ability.

Interestingly, more SCD also predicted increasing FC between the left dorsal anterior insula and dorsomedial prefrontal cortex. As the dorsal anterior insulae are components of the salience network, which modulates activity of the default mode and executive control networks [22], increased connectivity between the dorsal anterior insula and dorsomedial prefrontal cortex could reflect aberrant modulation of the default mode network at wakeful rest in SCD. While FC between left dorsal anterior insula and dorsomedial prefrontal cortex increased with greater degree of SCD that does not necessarily mean that the dorsal anterior insula has an excitatory role in prefrontal activity. Indeed, prior analyses have suggested that the dorsal anterior insula suppresses default mode network activity [61]. Increasing FC between the left dorsal anterior insula and dorsomedial prefrontal cortex for individuals reporting a greater degree of SCD could indicate decreased suppressive signaling to the default mode network. This may indicate that part of the experience of cognitive decline may reflect reduced ability to limit the self-referential, memory, and future planning mentation of the default mode network across scenarios that do not require this processing, i.e., components of the default mode network may remain too active during outward cognitive tasks that would normally result in default mode suppression and executive control recruitment via the salience network.

We also observed increasing FC between the dorsal anterior insula and orbitofrontal cortex in SCD. Though insular structural connectivity in humans remains largely unknown [22], tracer studies have established efferent projection from the insula to the lateral orbitofrontal cortex in the Old World Monkey [62] suggesting that this direct structural connectivity could exist in humans. As the lateral orbitofrontal cortex is involved in selecting context-appropriate behavior, decision-making, reward and value-based processing, as well as emotion regulation [63–65], increasing intrinsic FC between the dorsal anterior insula and lateral orbitofrontal cortex in SCD could indicate inappropriate recruitment of brain regions important for executive functions at wakeful rest in SCD. Furthermore, increased connectivity between the dorsal anterior insula and dorsomedial prefrontal cortex and between the dorsal anterior insula and orbitofrontal cortex could also reflect accelerated network dedifferentiation that could underlie the experience of SCD. As network dedifferentiation occurs in healthy aging and associates with poorer memory ability [66], accelerated dedifferentiation could be responsible for inefficient network processing and modularity that could influence the experience of decline.

We also observed that more SCD predicted decreasing FC between the left hippocampus and calcarine sulcus and lingual gyrus. As the lingual gyrus is involved in encoding visual memories [67, 68], and the hippocampus is important for memory encoding and retrieval [69], disrupted FC between these regions could underly poorer visual memory processing that influences self-report of decline. The calcarine sulcus is the location of primary visual cortex V1 [70] which may be influenced by healthy aging [71]. However, it remains unclear what decreasing FC between the primary visual cortex and hippocampus implies for SCD, especially as direct connectivity between medial the temporal regions and visual cortex is further along in the ventral and dorsal processing streams [72]. Nevertheless, decreasing FC between the hippocampus and calcarine sulcus/lingual gyrus may reflect disrupted information flow to the hippocampus. In addition, AD is extremely heterogenous but cortical atrophy in visual regions may reflect a subtype of AD related to poor visuospatial and executive functioning [52]. Thus, decreasing hippocampal to occipital FC in SCD, in addition to default mode network, strengthens support for SCD as a dementia precursor.

In the present analysis, degree of SCD did not predict baseline cognitive performance nor longitudinal change in visual working memory or episodic memory. By definition, SCD denotes perceived but uncorroborated cognitive decline [1]; therefore, it is not entirely surprising that SCD did not associate with change in cognitive performance, and a data collection period spanning 3 years may not have been enough time to capture subtle differences in cognitive trajectory between healthy aging and SCD. Furthermore, test-retest effects may have contributed to stability in cognitive performance. Though SCD associates with future decline [28], perceived decline can last many years before objective deficit [73]. Furthermore, although meta-analytic evaluation has determined that SCD status associates with lower cognitive performance [27], the effect size was small and sample differences can reflect variation within a normal performance range. Future research over a longer period of time should determine the point where cognitive performance trajectories for individuals with and without SCD diverge. Regardless, the lack of significant cognitive results in the present analysis alongside change in FC indicate that brain changes that relate to the experience of decline may precede detectable cognitive change. Thus, neuroimaging may be a sensitive method to detect incipient dementia.

The participants of this study were predominantly African American and female. We consider this a strength as many pre-dementia samples are predominantly white, and it remains unclear if results from those samples generalize across racial identity and ethnicity. Our findings suggest that they may, as our results are in line with previous observations. Nevertheless, larger and more diverse datasets are needed to determine generalizable brain changes related to SCD, and brain changes in SCD that may be influenced by sex, racial identity, or life experience.

### Limitations

A limitation of the current study is sampling error. As the sample was relatively small, model χ^2^ was smaller than model degrees of freedom for many voxels, which forced RMSEA to 0 by default. Furthermore, the 95% confidence intervals for fit indices were large. Therefore, the reliability of the parameter estimates reported here is low and requires verification from an analysis with a larger sample less influenced by sampling error. Nevertheless, the observed patterns of change involve connections between regions of the default mode network, which is affected by both healthy aging and dementia [58], and supports SCD as transition between healthy aging and dementia. Therefore, we believe that the results are meaningful to report as a preliminary exploration of functional connectivity change in SCD. Furthermore, separate evaluation of the left and right hippocampus produced similar patterns of results. This consistency lends additional confidence.

Another issue with the conditional latent growth models was that variability in latent slope was not significant at the α = .05 level for many voxels in the unconditional latent growth models. The magnitude of interindividual variability in FC change across voxels was actually large and potentially meaningful in the present analysis; however, our confidence in these values for interindividual variability is low because of the smaller sample size. Thus, while SCD explained variability in FC change, the effects may be small in the population at large. Regardless, the amount of interindividual variability in FC change we report for the unconditional growth models is plausible. Here, FC values were Fisher-Z-transformed Pearson correlations of brain signals, which in practice could result in FC values of ± .6. Thus, a variance in linear, 18-month change of .01, as was the case for variability in FC change between the hippocampus and dorsomedial prefrontal cortex, is large for a typical range of FC values. Thus, the range of slope variances of the sample may reflect plausible values, but the smaller dataset and accompanying sampling error make the analysis underpowered to label the variability as significant. Future work with larger datasets should establish reasonable expectations for variance in FC change.

## Conclusion

Subjective cognitive decline is a putative dementia precursor marked by perceived deficit in cognitive ability uncorroborated by formal assessment. The present analysis observed that a greater degree of SCD self-report predicted decreasing FC between components of the default mode network and increasing FC between salience and default mode network components. Greater network dedifferentiation in SCD may reflect an “accelerated” aging of the brain. In addition, this study provides additional support that SCD may be a precursor for dementia as subtle brain changes similar to those observed in AD were observed here. FC changes in the absence of cognitive changes suggest that brain alterations that underly the experience of decline, and could reflect the progression of incipient dementia, may emerge before cognitive assessment is sensitive enough to detect objective deficit.

## Supplementary Information

**Additional file 1: Supplementary Table 1.** Degree of subjective cognitive decline was stable over time. Here, we evaluated the effects of time and baseline subjective cognitive decline status on the MFQ Frequency of Forgetting subscale with a random intercepts mixed effects model. Number of observations = 129, N = 69. Here, SCD status was a binary variable that indicated whether a participant had significant worry about their cognitive faculties and sought medical advice prior to participation. While SCD status associated with greater MFQ-FoF, there was no appreciable overall effect of time, or a significant time by SCD status interaction effect on MFQ-FoF. From this result, we decided to use baseline MFQ-FoF as a covariate in subsequent models rather than include MFQ-FoF as a time-varying covariate. **Supplementary Table 2.** Descriptive statistics for MFQ-FoF across all measurements. **Supplementary Table 3.** Mean ± standard deviation for absolute and relative framewise displacement in millimeters across participants across all measurements. **Supplementary Table 4.** Random intercepts mixed effects model evaluating the effects of degree of SCD (via MFQ-FoF), measurement occasion, and their interaction on Mini-Mental State Examination performance. MFQ-FoF, did not associate with baseline Mini-Mental State Examination score, *r* = -.05, *p* = .70, and explained < .01% of variance when controlling for age, *p* = .99. Furthermore, evaluation of all timepoints with a random intercepts mixed model revealed no main effects of MFQ-FoF nor measurement occasion on Mini-Mental State Examination score. There was no significant interaction between MFQ-FoF or measurement occasion. **Supplementary Table 5.** Model fit indices for the latent growth models as well as unstandardized and standardized parameter estimates for the peak voxels of the significant FC clusters. P-values reflect the unstandardized model. Single tildes (~) represent a regression while double tildes (~~) represent a (residual) variance or covariance. A single tilde followed by a 1 represents an intercept. **Supplementary Table 6.** Latent growth curve model evaluating the effect of degree of SCD and age on the linear change in Wechsler Memory Scale IV Visual Working Memory Index performance. Single tildes represent a regression while double tildes represent a (residual) variance. A single tilde followed by a 1 represents an intercept. Working memory slope variance fixed to 0. **Supplementary Table 7.** Latent growth curve model evaluating the effect of MFQ-FoF and age on linear change in WMS-IV Delayed Memory performance. Single tildes represent a regression while double tildes represent a (residual) variance or a covariance. A single tilde followed by a 1 represents an intercept.

## Data Availability

The datasets analyzed for the current study are not publicly available but are available from the corresponding author on reasonable request. Example code for running longitudinal conditional latent growth structural equation models can be found at: https://github.com/rviviano/data-tools/tree/master/recipes/neuropointillist.

## Abbreviations

AD: Alzheimer’s disease
ICA-AROMA: Independent Component Analysis Automatic Removal of Motion Artifacts
CFI: Comparative Fit Index
FC: Functional connectivity
FIML: Full information maximum likelihood
FOV: Field of View
FWHM: Full width at half maximum
MFQ-FoF: Memory Functioning Questionnaire Frequency of Forgetting Subscale
RMSEA: Root mean square error of approximation
SCD: Subjective cognitive decline
SRMR: Standardized root mean square residual
TE: Echo time
TR: Repetition time
WASI: Wechsler Abbreviated Scale of Intelligence
WMS-IV: Wechsler Memory Scale IV
