# Supplementary material for: Longitudinal change in hippocampal and dorsal anterior insulae functional connectivity in subjective cognitive decline
Source: Alzheimers Res Ther. 2021 May 31;13:108. doi: 10.1186/s13195-021-00847-y (PMC8166120; doi:10.1186/s13195-021-00847-y)
Supplement: Supplementary file 1 — Additional file 1: Supplementary Table 1. Degree of subjective cognitive decline was stable over time. Here, we evaluated the effects of time and baseline subjective cognitive decline status on the MFQ Frequency of Forgetting subscale with a random intercepts mixed effects model. Number of observations = 129, N = 69. Here, SCD status was a binary variable that indicated whether a participant had significant worry about their cognitive faculties and sought medical advice prior to participation. While SCD status associated with greater MFQ-FoF, there was no appreciable overall effect of time, or a significant time by SCD status interaction effect on MFQ-FoF. From this result, we decided to use baseline MFQ-FoF as a covariate in subsequent models rather than include MFQ-FoF as a time-varying covariate. Supplementary Table 2. Descriptive statistics for MFQ-FoF across all measurements. Supplementary Table 3. Mean ± standard deviation for absolute and relative framewise displacement in millimeters across participants across all measurements. Supplementary Table 4. Random intercepts mixed effects model evaluating the effects of degree of SCD (via MFQ-FoF), measurement occasion, and their interaction on Mini-Mental State Examination performance. MFQ-FoF, did not associate with baseline Mini-Mental State Examination score, r = -.05, p = .70, and explained < .01% of variance when controlling for age, p = .99. Furthermore, evaluation of all timepoints with a random intercepts mixed model revealed no main effects of MFQ-FoF nor measurement occasion on Mini-Mental State Examination score. There was no significant interaction between MFQ-FoF or measurement occasion. Supplementary Table 5. Model fit indices for the latent growth models as well as unstandardized and standardized parameter estimates for the peak voxels of the significant FC clusters. P-values reflect the unstandardized model. Single tildes (~) represent a regression while double tildes (~~) represent a (residual) va [file 13195_2021_847_MOESM1_ESM.docx]

**Supplemental Materials**

|  | Random Effects | | Fixed Effects | | | ANOVA table | |
| --- | --- | --- | --- | --- | --- | --- | --- |
|  | Variance | Std. Dev. | Estimate | Std. Error | T Value | Mean Sq. | F Value |
| Subject | .30 | .55 |  |  |  |  |  |
| Residual | .39 | .62 |  |  |  |  |  |
| Intercept |  |  | 2.72 | .18 | 14.99 |  |  |
| Time |  |  | -.03 | .09 | -.34 | .19 | .50 |
| SCD |  |  | .93 | .31 | 3.03 | 9.43 | 24.49 |
| Time × SCD |  |  | -.01 | .16 | -.09 | .00 | .01 |

Supplementary Table 1. Degree of subjective cognitive decline was stable over time. Here, we evaluated the effects of time and baseline subjective cognitive decline status on the MFQ Frequency of Forgetting subscale with a random intercepts mixed effects model. Number of observations = 129, N = 69. Here, SCD status was a binary variable that indicated whether a participant had significant worry about their cognitive faculties and sought medical advice prior to participation. While SCD status associated with greater MFQ-FoF, there was no appreciable overall effect of time, or a significant time by SCD status interaction effect on MFQ-FoF. From this result, we decided to use baseline MFQ-FoF as a covariate in subsequent models rather than include MFQ-FoF as a time-varying covariate.

|  | Time 1 (N = 69) | Time 2 (N = 34) | Time 3 (N = 28) |
| --- | --- | --- | --- |
| Mean | 2.99 | 2.95 | 2.91 |
| St.Dev | .91 | .95 | .79 |
| Min | 1.21 | 1.15 | 1.24 |
| Max | 4.97 | 5.06 | 5.24 |
| Range | 3.76 | 3.91 | 4.00 |

Supplementary Table 2. Descriptive statistics for MFQ-FoF across all measurements.

|  | Time 1 (N = 69) | Time 2 (N = 34) | Time 3 (N = 28) |
| --- | --- | --- | --- |
| Absolute FD | .43 ± .29 | .37 ± .18 | .35 ± .20 |
| Relative FD | .17 ± .07 | .17 ± .08 | .17 ± .08 |

Supplementary Table 3. Mean ± standard deviation for absolute and relative framewise displacement in millimeters across participants across all measurements.

| Mini Mental State Examination Random Intercepts Model | | | | | | | | | |
| --- | --- | --- | --- | --- | --- | --- | --- | --- | --- |
|  | Random Effects | | Fixed Effects | | | 95% CI | | ANOVA table | |
|  | Variance | Std. Dev. | Estimate | Std. Error | T Value | 2.5% | 97.5% | Mean Sq. | F |
| Subject | 1.23 | 1.11 |  |  |  |  |  |  |  |
| Residual | 1.61 | 1.27 |  |  |  |  |  |  |  |
| Intercept |  |  | 28.58 | .49 | 58.84 | 27.72 | 29.55 |  |  |
| Time |  |  | .24 | .35 | .70 | -.40 | .88 | 1.31 | .81 |
| MFQ |  |  | -.07 | .22 | -.33 | -.53 | .30 | .46 | .29 |
| Time × MFQ | |  | -.05 | .16 | -.34 | -.33 | .24 | .18 | .11 |

Supplementary Table 4. Random intercepts mixed effects model evaluating the effects of degree of SCD (via MFQ-FoF), measurement occasion, and their interaction on Mini-Mental State Examination performance. MFQ-FoF, did not associate with baseline Mini-Mental State Examination score, *r* = -.05, *p* = .70, and explained < .01% of variance when controlling for age, *p* = .99. Furthermore, evaluation of all timepoints with a random intercepts mixed model revealed no main effects of MFQ-FoF nor measurement occasion on Mini-Mental State Examination score. There was no significant interaction between MFQ-FoF or measurement occasion.

|  | Left HC - DMPFC | | | Left HC - Visual | | | Right HC - DMPFC | | | Left Insula - DMPFC | | | Left Insula - OFC | | |
| --- | --- | --- | --- | --- | --- | --- | --- | --- | --- | --- | --- | --- | --- | --- | --- |
| Parameter | Est. | (Std) | P | Est. | (Std) | P | Est. | (Std) | P | Est. | (Std) | P | Est. | (Std) | P |
| *χ^2^*(3) | 3.25 |  | .36 | 2.20 |  | .53 | 3.36 |  | .34 | .40 |  | .94 | .30 |  | .96 |
| CFI | .99 |  |  | 1.00 |  |  | .97 |  |  | 1.00 |  |  | 1.00 |  |  |
| RMSEA | .04 |  |  | .00 |  |  | .04 |  |  | .00 |  |  | .00 |  |  |
| RMSEA Lower | .00 |  |  | .00 |  |  | .00 |  |  | .00 |  |  | .00 |  |  |
| RMSEA Upper | .21 |  |  | .18 |  |  | .21 |  |  | .04 |  |  | .00 |  |  |
| SRMR | .06 |  |  | .05 |  |  | .07 |  |  | .02 |  |  | .02 |  |  |
| FC I ~ 1 | -.04 | (-.39) | .41 | **-.12** | **(-1.34)** | **.00** | -.03 | (-.31) | .48 | .07 | (.53) | .18 | .05 | (.64) | .19 |
| FC I ~~ I | .01 | (.87) | .09 | .00 | (.59) | .45 | .01 | (.89) | .15 | .01 | (.77) | .06 | .01 | (.77) | .39 |
| FC S ~ 1 | **.12** | **(1.56)** | **.00** | **.09** | **(1.42)** | **.00** | **.08** | **(1.30)** | **.00** | **-.13** | **(-2.70)** | **.00** | **-.11** | **(-1.55)** | **.00** |
| FC S ~~ S | .00 | (.49) | .37 | .00 | (.55) | .63 | .00 | (.46) | .53 | .00 | (.09) | .96 | .00 | (.34) | .57 |
| FC I ~~ S | -.00 | (-.64) | .29 | -.00 | (-.16) | .90 | -.00 | (-.80) | .35 | -.00 | (-1.21) | .61 | -.00 | (-.73) | .55 |
| FC I ~ MFQ | **.04** | **(.36)** | **.03** | **.05** | **(.54)** | **.00** | .03 | (.27) | .09 | **-.07** | **(-.48)** | **.00** | **-.04** | **(-.47)** | **.02** |
| FC S ~ MFQ | **-.06** | **(-.69)** | **.00** | **-.05** | **(-.67)** | **.00** | **-.05** | **(-.71)** | **.00** | **.05** | **(.96)** | **.00** | **.06** | **(.76)** | **.00** |
| FC I ~ Age | -.00 | (-.15) | .50 | **-.00** | **(-.42)** | **.01** | -.00 | (-.23) | .14 | .00 | (.06) | .68 | -.00 | (-.03) | .86 |
| FC S ~ Age | -.00 | (-.12) | .51 | .00 | (.18) | .26 | -.00 | (-.12) | .54 | -.00 | (-.12) | .67 | .00 | (.19) | .26 |

Supplementary Table 5. Model fit indices for the latent growth models as well as unstandardized and standardized parameter estimates for the peak voxels of the significant FC clusters. P-values reflect the unstandardized model. Single tildes (~) represent a regression while double tildes (~~) represent a (residual) variance or covariance. A single tilde followed by a 1 represents an intercept.

| Parameter | Est. | (Std) | P |
| --- | --- | --- | --- |
| *χ^2^* | 2.77 |  | .60 |
| DF | 4.00 |  |  |
| CFI | 1.00 |  |  |
| RMSEA | .00 |  |  |
| RMSEA Lower | .00 |  |  |
| RMSEA Upper | .16 |  |  |
| SRMR | .03 |  |  |
| WrkMem I ~ 1 | .40 | (3.95) | .00 |
| WrkMem I ~~ I | .01 | (.60) | .00 |
| WrkMem S ~ 1 | -.00 | (-.06) | .98 |
| WrkMem S ~~ S | .00 | (.00) | NA |
| WrkMem I ~~ S | .00 | (NA) | .26 |
| WrkMem I ~ MFQ-FoF | -.01 | (-.04) | .70 |
| WrkMem S ~ MFQ-FoF | -.00 | (-.66) | .56 |
| WrkMem I ~ Age | -.01 | (-.62) | .00 |
| WrkMem S ~ Age | -.00 | (-.67) | .49 |

Supplementary Table 6. Latent growth curve model evaluating the effect of degree of SCD and age on the linear change in Wechsler Memory Scale IV Visual Working Memory Index performance. Single tildes represent a regression while double tildes represent a (residual) variance. A single tilde followed by a 1 represents an intercept. Working memory slope variance fixed to 0.

| Parameter | Est. | (Std) | P |
| --- | --- | --- | --- |
| *χ^2^* | 4.35 |  | .23 |
| DF | 3.00 |  |  |
| CFI | .99 |  |  |
| RMSEA | .08 |  |  |
| RMSEA Lower | .00 |  |  |
| RMSEA Upper | .22 |  |  |
| SRMR | .06 |  |  |
| DelMem I ~ 1 | .44 | (3.75) | .00 |
| DelMem I ~~ I | .01 | (.76) | .00 |
| DelMem S ~ 1 | .02 | (.47) | .07 |
| DelMem S ~~ S | .00 | (.89) | .26 |
| DelMem I ~~ S | -.00 | (-.44) | .31 |
| DelMem I ~ MFQ-FoF | .01 | (.06) | .56 |
| DelMem S ~ MFQ-FoF | -.00 | (-.06) | .52 |
| DelMem I ~ Age | -.01 | (-.50) | .00 |
| DelMem S ~ Age | -.00 | (-.32) | .00 |

Supplementary Table 7. Latent growth curve model evaluating the effect of MFQ-FoF and age on linear change in WMS-IV Delayed Memory performance. Single tildes represent a regression while double tildes represent a (residual) variance or a covariance. A single tilde followed by a 1 represents an intercept.
